# Activated Drp1 Initiates the Formation of Endoplasmic Reticulum‐Mitochondrial Contacts via Shrm4‐Mediated Actin Bundling

**DOI:** 10.1002/advs.202304885

**Published:** 2023-11-01

**Authors:** Chenyang Duan, Ruixue Liu, Lei Kuang, Zisen Zhang, Dongyao Hou, Danyang Zheng, Xinming Xiang, He Huang, Liangming Liu, Tao Li

**Affiliations:** ^1^ Department of Shock and Transfusion State Key Laboratory of Trauma Burns and Combined Injury Daping Hospital Army Medical University Chongqing 400042 P. R. China; ^2^ Department of Anesthesiology The Second Affiliated Hospital of Chongqing Medical University Chongqing 400010 P. R. China

**Keywords:** actin bundling, Drp1, ER‐Mito contact, mitochondrial fission, shrm4

## Abstract

Excessive mitochondrial fission following ischemia and hypoxia relies on the formation of contacts between the endoplasmic reticulum and mitochondria (ER‐Mito); however, the specific mechanisms behind this process remain unclear. Confocal microscopy and time course recording are used to investigate how ischemia and hypoxia affect the activation of dynamin‐related protein 1 (Drp1), a protein central to mitochondrial dynamics, ER‐Mito interactions, and the consequences of modifying the expression of Drp1, shroom (Shrm) 4, and inverted formin (INF) 2 on ER‐Mito contact establishment. Both Drp1 activation and ER‐Mito contact initiation cause excessive mitochondrial fission and dysfunction under ischemic‐hypoxic conditions. The activated form of Drp1 aids in ER‐Mito contact initiation by recruiting Shrm4 and promoting actin bundling between the ER and mitochondria. This process relies on the structural interplay between INF2 and scattered F‐actin on the ER. This study uncovers new roles of cytoplasmic Drp1, providing valuable insights for devising strategies to manage mitochondrial imbalances in the context of ischemic‐hypoxic injury.

## Graphical abstract

Activated Drp1 Initiates the formation of endoplasmic reticulum‐mitochondrial contacts via Shrm4‐Mediated Actin Bundling under ischemic‐hypoxic conditions, Drp1 activation and ER‐Mito contact initiation precede excessive mitochondrial fission and dysfunction. Activated Drp1 recruits Shrm4, promotes actin bundling between the ER and mitochondria, and relies on INF2's interaction with F‐actin on the ER. This study elucidates novel functions of cytoplasmic Drp1, offering invaluable insights for addressing the mitochondrial imbalance in ischemic‐hypoxic injury.

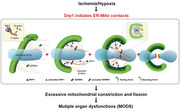

## 1 Introduction

Mitochondria serve as the energy‐processing units of eukaryotic cells. They maintain a dynamic balance between continuous fission and fusion under normal conditions. However, pathological stimuli, such as ischemia and hypoxia, disrupt this balance, leading to excessive mitochondrial fission and cell damage.^[^ ^1^, ^2^ ^]^ Numerous studies have demonstrated the close relationship between excessive mitochondrial fission and various diseases, including shock,^[^ ^3^ ^]^ sepsis,^[^ ^4^ ^]^ COVID‐19,^[^ ^5^ ^]^ neurodegeneration,^[^ ^6^ ^]^ ischemic diseases, and aging.^[^ ^7^ ^]^ Hence, elucidation of the associated mechanism will inform the development of measures to protect mitochondrial quality and treat diseases related to mitochondrial injury.

Dynamin‐related protein 1 (Drp1) is the main protein involved in mitochondrial fission. However, mitochondrial fission requires a series of processes, including the translocation of DRP1 from the cytoplasm to the mitochondria and the cleavage of mitochondrial membranes via the GTPase activity of Drp1.^[^ ^2^ ^]^ Before mitochondrial fission, the endoplasmic reticulum (ER) must wrap around the mitochondria to create ER‐mitochondrial contacts (ER‐Mito contacts), leading to mitochondrial constriction. Drp1 is then passively recruited to the ER‐Mito contact sites to facilitate mitochondrial fission.^[^ ^8^, ^9^ ^]^ Moreover, ER‐Mito contact formation primarily relies on inverted formin‐2 (INF2), which is crucial in polymerizing G‐actin into F‐actin.^[^ ^9^ ^]^ Meanwhile, no studies have established the function of cytoplasmic Drp1 before recruitment to the mitochondria. It is also unclear whether Drp1 participates in ER‐Mito contact formation, or whether it has additional functions beyond its role in mitochondrial fission following recruitment to the ER‐Mito contact site. Additionally, the mechanism by which F‐actin links the ER to the mitochondria to form contacts is not fully understood.

In this study, we investigate the underlying mechanism of Drp1 in the formation of ER‐Mito contact and mitochondrial constriction. To this end, we employ various experimental models, including vascular smooth muscle cells (VSMCs) transfected with Drp1, Shrm4, and INF2 overexpression or deletion plasmids, Drp1‐knockout mice (Drp1+/‐), and a Sprague‐Dawley rat model of ischemia. Results suggest that Drp1 has a broader role than previously reported. That is, it is not only responsible for mediating mitochondrial fission but also acts as a critical regulator of ER‐Mito contact formation and mitochondrial constriction, the two preconditions of excessive mitochondrial fission following ischemia and hypoxia. Our findings highlight novel functions of cytoplasmic Drp1 and provide valuable insights for preventing and treating mitochondrial quality imbalances in the context of ischemic‐hypoxic injury.

## 2 Results

### 2.1 Participation of Activated Cytoplasmic DRP1 in the Formation of ER‐Mito Contacts Before Translocation to Mitochondria to Mediate Mitochondrial Fission

**Figure 1:**
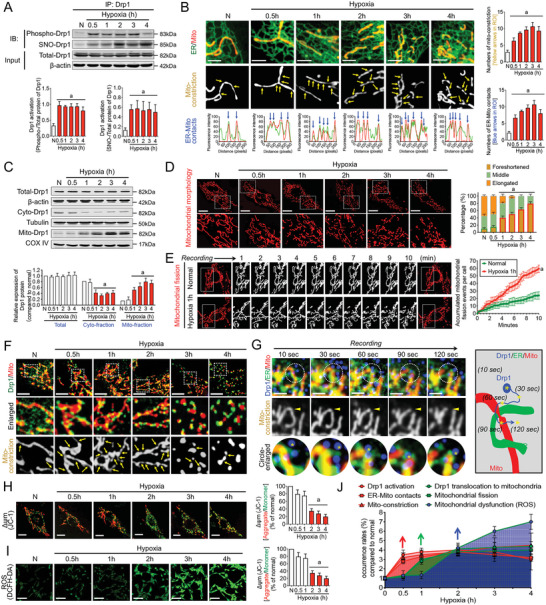
Time course observation of Drp1 activation, ER‐Mito contact formation, mitochondrial fission, and mitochondrial functions in VSMCs under extended hypoxia. A) Co‐IP results showing Drp1 activation in VSMCs with the extension of hypoxia. phospho‐Drp1, Drp1 phosphorylation; SNO‐Drp1, Drp1 S‐nitrosylation. (*n* = 3 per group). B) ER‐Mito contacts labeled with ERtracker and Mitotracker in VSMCs with the extension of hypoxia. Yellow arrows: mitochondrial constriction. Blue arrows: ER‐Mito contacts. Mitochondrial constriction and ER‐Mito contacts were enumerated in the region of interest (ROI) by Image J. (*n* = 5 per group). C) Drp1 expression in total, cytoplasmic, and mitochondrial fractions of VSMCs with the extension of hypoxia. (*n* = 3 per group). D) Confocal images of mitochondrial morphology in VSMCs with the extension of hypoxia (bar, 25 µm). Quantitation is done in triplicate and scored into three categories: foreshortened, middle, and elongated mitochondria, with 100 cells scored per group. E) Time‐lapse images of mitochondrial morphologic alternations in VSMCs every 15 s after 1 h hypoxia determined by confocal immuno‐staining (bar, 25 µm). Images correspond to the selected area visualized from the same viewpoint, and quantitative data are shown (*n* = 25–30 cells per group). F) Co‐localization of GFP‐labeled Drp1 and Mitotracker‐labeled mitochondria with the extension of hypoxia (bar, 5 µm). Yellow arrows: mitochondrial constriction. G) Time course recording of ER, mitochondria, and Drp1 motion tracking (the whole process from ER‐Mito gathering to mitochondria fission) in VSMCs labeled by BFP‐Drp1, ERtracker, and Mitotracker at 0.5 h hypoxia (bar, 5 µm). *Drp1 particles during the process; the tracking path is shown in the pattern image. H) Mitochondrial membrane potential labeled by JC‐1 monomer (green fluorescent probe) and JC‐1 aggregate (Red fluorescent probe) in VSMCs with the extension of hypoxia (bar, 25 µm). I) ROS production as reflected by DCFH‐DA fluorescence in VSMCs with the extension of hypoxia (bar, 100 µm). J) Time‐dynamic sketch of Drp1 activation, ER‐Mito contacts, mitochondrial constriction, Drp1 translocation to mitochondria, mitochondrial fission, and mitochondrial dysfunction (ROS accumulation) events with the extension of hypoxia. Red arrow: start of Drp1 activation, frequency of ER‐Mito contacts, and mitochondrial constriction after hypoxia. Green arrow: start of considerable Drp1 translocation to mitochondria and excessive mitochondrial fission after hypoxia. Blue arrow: start of mitochondrial dysfunction (ROS accumulation) after hypoxia. Red bar: time at which the indicator changes significantly. a: *p* < 0.05 compared with the normal group.

Our previous studies^[^ ^3^, ^10^, ^11^, ^12^, ^13^ ^]^ have demonstrated that during ischemia and hypoxia‐induced mitochondrial quality imbalance, various pathological processes occur, including the activation of DRP1, frequent formation of ER‐Mito contacts, excessive mitochondrial fission, and mitochondrial dysfunction. However, an in‐depth analysis of the temporal changes in these pathological processes is lacking. Herein, we conducted continuous time course observations using VSMCs, which are reportedly highly sensitive to mitochondrial quality changes following ischemia and hypoxia stimulation.^[^ ^12^ ^]^ Our results showed that Drp1 activity changed in the early stage of hypoxia (0.5 h), as evidenced by an increase in Drp1 phosphorylation and S‐nitrosylation modification compared to that in normal condition (*p* < 0.05; **Figure** **1**A). Moreover, the formation of ER‐Mito contacts and mitochondrial constriction increased following 0.5 h of hypoxia (*p* < 0.05; Figure 1B; Figure S1A, Supporting Information). These findings suggest that Drp1 activation is synchronous with the formation of ER‐Mito contacts.

Despite there being no significant change in the overall protein expression of Drp1 after hypoxia (*p* > 0.05), a significant decrease in cytoplasmic Drp1 content and an increase in mitochondrial Drp1 expression after hypoxia 1 h were observed (*p* < 0.05; Figure 1C). This suggests high levels of cytoplasmic Drp1 translocate to mitochondria following hypoxia 1 h. The confocal results verified that the co‐localization of Drp1 with mitochondria gradually increased following hypoxia 1 h (*p* < 0.05; Figure S1B, Supporting Information). Meanwhile, the continuous time course observation showed no significant change in the mitochondrial morphology at hypoxia 0.5 h (*p* > 0.05), whereas mitochondrial fragmentation occurred after hypoxia 1 h (*p* < 0.05; Figure 1D). Confocal video observation revealed that excessive mitochondrial fission primarily caused mitochondrial fragmentation at hypoxia 1 h (*p* < 0.05; Figure 1E; Movies S1 and S2, Supporting Information). Via simultaneous labeling of Drp1, ER, and mitochondria, we confirmed that excessive mitochondrial fission occurred after the formation of ER‐Mito contacts and mitochondrial constriction (Figure 1F). The continuous time course observation also revealed that cytoplasmic Drp1 first translocated to the ER within the first 10–30 s to participate in ER‐Mito contact formation and mitochondrial constriction. Within 30–60 s, a large amount of cytoplasmic Drp1 was further recruited to the ER‐Mito contact sites, and within 90–120 s, the mitochondrial fission process was completed (Figure 1G). These findings suggest that cytoplasmic Drp1 may initially translocate to the ER to participate in ER‐Mito contact formation before translocating to the mitochondria and mediating mitochondrial fission. This may account for why Drp1 becomes activated and ER‐Mito contacts form before cytoplasmic Drp1 translocates to the mitochondria and mitochondrial fission occurs.

Furthermore, we assessed changes in mitochondrial function following hypoxia. The reduction in mitochondrial membrane potential (Figure 1H; Figure S1C, Supporting Information) and accumulation of ROS content (Figure 1I) primarily began at the 2‐h mark after hypoxia, occurring later than the onset of excessive mitochondrial fission. These results confirm that the activation of Drp1 and ER‐Mito contact formation occurs earlier than the excessive mitochondrial fission and mitochondrial dysfunction associated with hypoxia‐induced mitochondrial quality imbalance. This may be attributed to activated cytoplasmic Drp1, which initiates the formation of ER‐Mito contacts (Figure 1J).

**Figure 2:**
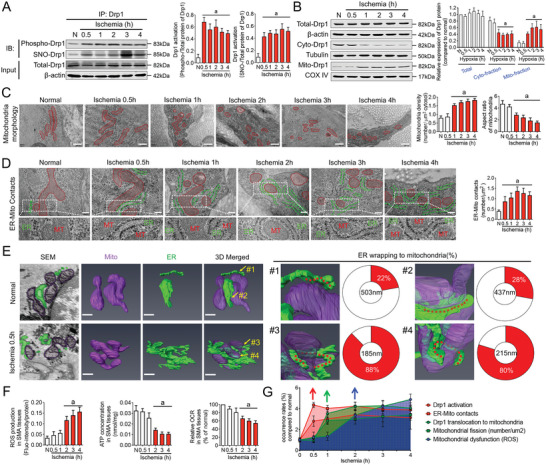
Time course observation of Drp1 activation, ER‐Mito contact formation, mitochondrial fission, and mitochondrial functions in VSMCs under extended ischemia. A) Co‐IP results showing Drp1 activation in VSMCs with the extension of ischemia. phospho‐Drp1, Drp1 phosphorylation; SNO‐Drp1, Drp1 S‐nitrosylation (*n* = 3 per group). B) Drp1 expression in total, cytoplasmic and mitochondrial fractions of VSMCs with the extension of ischemia. (*n* = 3 per group). C) Representative TEM images of mitochondrial morphology (red) in VSMCs with the extension of ischemia (bar, 500 nm). Mitochondrial density and aspect ratio (length/width) are calculated in each group (*n* = 5 per group). D) Representative TEM images of ER‐Mito contacts in VSMCs with the extension of ischemia (bar, 200 nm); red: mitochondria, green: ER. Number of ER‐Mito contacts are calculated for each group (*n* = 5 per group). E) The 3D reconstruction of ER (green) and mitochondria (purple) at contact sites in VSMCs imaged by FIB‐SEM. First column: 2D tomographs of ER‐Mito contacts (bar, 300 nm). ER wrapping is defined as regions where the ER membrane localized within 30 nm of the mitochondrial membrane. Right: percentage of the mitochondrial circumference that contacts the ER membrane [red is contact, white is not]. Center: diameter (nm) of each mitochondrion at positions of ER contact. F) Mitochondrial functions including ROS production, ATP concentration, and mitochondrial respiration (OCR) in VSMCs from superior mesenteric artery (SMA) tissue with the extension of ischemia. G) Time‐dynamic sketch of Drp1 activation, ER‐Mito contact formation, Drp1 translocation to mitochondria, mitochondrial fission, and mitochondrial dysfunction (ROS accumulation) with the extension of ischemia. Red arrow: start of Drp1 activation and frequency of ER‐Mito contacts after ischemia. Green arrow: start of large amounts of Drp1 translocating to mitochondria and excessive mitochondrial fission after ischemia. Blue arrow: start of mitochondrial dysfunctions (ROS accumulation) after ischemia. Red bar: time point at which the indicator significantly changes. a: *p* < 0.05 compared with the normal group.

To investigate temporal changes in Drp1 activation, ER‐Mito contacts, mitochondrial fission, and mitochondrial function in vivo, we examined the modification and translocation of Drp1 in vascular tissue following ischemic injury. The results indicated that phosphorylation and S‐nitrosylation modifications of Drp1 significantly increased after 0.5 h of ischemia (*p* < 0.05; **Figure** **2**A), which preceded the translocation of Drp1 from the cytoplasm to the mitochondria (*p* < 0.05; Figure 2B). These findings are consistent with the results observed at the cellular level. Transmission electron microscopy (TEM) observations revealed that the increase in the abundance of fragmented mitochondria primarily began 1 h after ischemia (*p* < 0.05; Figure 2C; Figure S2A, Supporting Information), whereas ER wrapping of mitochondria occurred 0.5 h after ischemia (*p* < 0.05; Figure 2D; Figure S2B, Supporting Information). To rule out the possibility of chance observations of ER‐Mito contact sections, we performed a three‐dimensional reconstruction of ER‐Mito contact using FIB‐SEM (Movies S3 and S4, Supporting Information). Under normal conditions, the winding areas of the ER and mitochondria were only 22% (#1) and 28% (#2), respectively, and the mitochondrial diameter was 503 nm (#1) and 437 nm (#2). However, at ischemia 0.5 h, the winding areas of ER and mitochondria reached 88% (#3) and 80% (#4), respectively, and the diameter of the mitochondria at the ER‐Mito contact site was significantly reduced (# 3:185 nm; # 4:215 nm; Figure 2E). These results confirm that Drp1 activation and ER‐Mito contact formation occur earlier than the translocation of cytoplasmic Drp1 to the mitochondria and the occurrence of mitochondrial fission in vivo.

### 2.2 Drp1‐Initiated ER‐Mito Contact: Prerequisite for Excessive Mitochondrial Fission and Dysfunctions, Possibly Involving Shrm4

To investigate the role of Drp1 in forming ER‐Mito contacts and mitochondrial constriction after ischemia and hypoxia, we simultaneously labeled the ER and mitochondria in VSMCs, and deleted Drp1 (Figure S3A, Supporting Information). The confocal microscopy results demonstrated that the increased formation of ER‐Mito contacts induced by hypoxia was significantly reduced after Drp1 deletion (*p* < 0.05; Figure S3B, Supporting Information).

**Figure 3:**
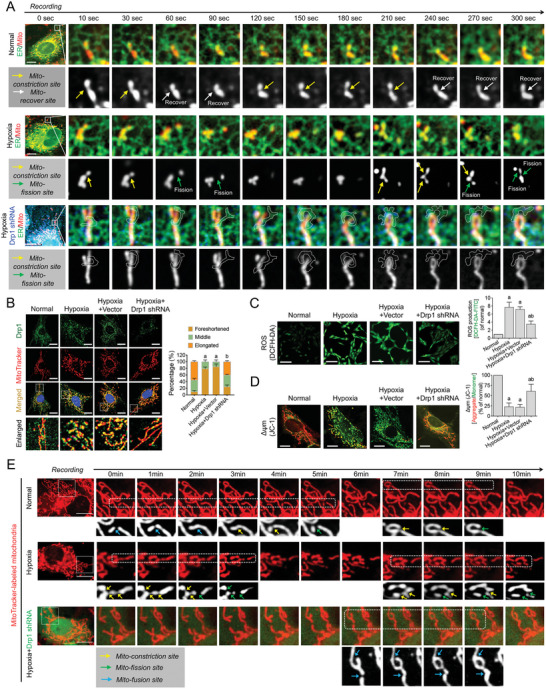
Influence of Drp1 deletion on ER‐Mito contact formation, mitochondrial fission, and mitochondrial functions in VSMCs after hypoxia. A) Time course recording the effect of Drp1 deletion (BFP‐labeled Drp1 shRNA) on the motions of GFP‐labeled ER and RFP‐labeled mitochondria in VSMCs after hypoxia (bar, 25 µm). Yellow arrows: mitochondrial constriction site. White arrows: site of mitochondrial recovery from constriction. Green arrows: mitochondrial fission site. B) Co‐localization of Drp1 and mitochondria in hypoxia‐induced VSMCs after Drp1 deletion (bar, 25 µm). Quantitation is done in triplicate and scored into three categories: foreshortened, middle, and elongated mitochondria, with 100 cells scored per group. C) ROS production as reflected by DCFH‐DA fluorescence in hypoxia‐induced VSMCs after DRP1 deletion (bar, 100 µm). D) Mitochondrial membrane potential labeled by JC‐1 monomer (green fluorescent probe) and JC‐1 aggregate (red fluorescent probe) in hypoxia‐induced VSMCs after Drp1 deletion (bar, 25 µm). E) Time course recording the mitochondrial dynamics, including fission and fusion events, in hypoxia‐induced VSMCs after Drp1 deletion (bar, 25 µm). Yellow arrows: mitochondrial constriction site. Green arrows: mitochondrial fission site. Blue arrows: mitochondrial fusion site. a: *p* < 0.05 compared with the normal group. b: *p* < 0.05 compared with the hypoxia group.

Confocal video observations showed that, under normal conditions, mitochondrial constriction occurred infrequently, and was not sufficiently strong to induce mitochondrial fission. This may be because the traction force generated by actin between the ER and mitochondria was insufficient to cause complete wrapping of the ER around the mitochondria.^[^ ^14^ ^]^ Under hypoxic conditions, the constriction of mitochondria induced by ER‐Mito contact rapidly promoted the occurrence of mitochondrial fission. Meanwhile, Drp1 deletion significantly reduced ER‐Mito contacts and transformed the interaction between the ER and mitochondria into a relative sliding movement (**Figure** **3**A). These results suggest that Drp1 may participate in the regulation of the actin‐mediated traction anchoring process between the ER and mitochondria.

After Drp1 deletion, we observed a significant reduction in the co‐localization of Drp1 and mitochondria induced by hypoxia (*p* < 0.05; Figure 3B), the restoration of mitochondrial morphology from punctate to elongated (*p* < 0.05; Figure S3C, Supporting Information), a significant decrease in mitochondrial ROS accumulation (*p* < 0.05; Figure 3C), and a significant increase in mitochondrial membrane potential (*p* < 0.05; Figure 3D). Confocal long‐term video recording revealed that under normal conditions, mitochondria were in a dynamically balanced state of fission and fusion. After hypoxia, the frequency of mitochondrial constriction and fission increased significantly. However, following DRP1 deletion, the constriction and fission of mitochondria induced by ER‐Mito contact were significantly reduced (Figure 3E). These findings confirm that Drp1‐mediated ER‐Mito contact is a crucial factor for excessive mitochondrial fission and mitochondrial dysfunction in vitro.

**Figure 4:**
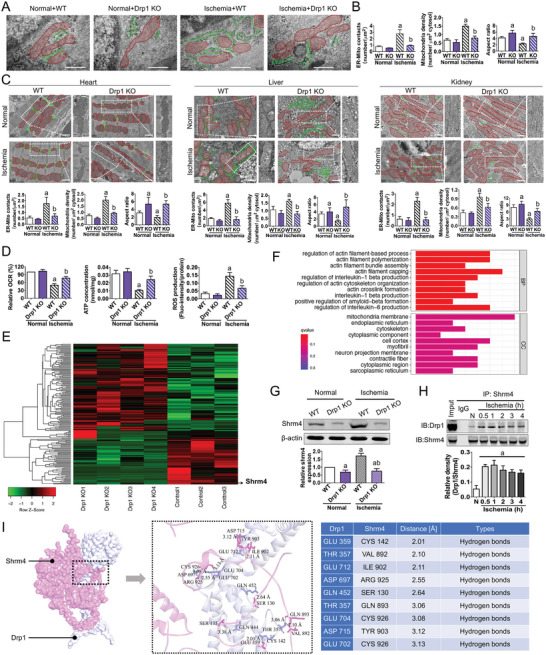
Changes in ER‐Mito contact formation, mitochondrial fission, and mitochondrial functions in Drp1‐knockout mice after ischemia. A) Representative TEM images of ER‐Mito contacts in SMAs from wild‐type and Drp1‐knockout mice in normal and ischemia conditions (bar, 500 nm). Mitochondria are labeled in red and ERs are labeled in green. B) Statistical data of ER‐Mito contacts, mitochondrial density, and aspect ratio (length/width) in TEM images of SMAs from wild‐type and Drp1‐knockout mice in normal and ischemic conditions. C) Representative TEM images of ER‐Mito contacts in heart, liver, and kidney tissues from wild‐type and Drp1‐knockout mice in normal and ischemic conditions (bar, 1 µm). Mitochondria are labeled in red and ERs are labeled in green. Statistical data of ER‐Mito contacts, mitochondrial density, and aspect ratio (length/width) are shown. D) Various mitochondrial functions, including mitochondrial respiration (OCR), ATP concentration, and ROS accumulation in SMAs from wild‐type and Drp1‐knockout mice in normal and ischemic conditions. E,F) Transcriptional heatmap and GO enrichment analysis of differentially expressed genes between wild‐type and Drp1‐knockout mice (data update: GSE124096). G) Western blotting results of Shrm4 protein abundance in SMAs from wild‐type and Drp1‐knockout mice in normal and ischemic conditions (*n* = 3 per group). H) Representative Co‐IP assay of Drp1‐Shrm4 interactions in SMAs with the extension of ischemia (*n* = 3 per group). I) Structural analysis of Drp1 and Shrm4. White: Drp1 structure; Pink:Shrm4 structure. The full length structural formulas of Drp1 and Shrm4 are derived from AlphaFold. Purple bar: Drp1‐knockout group. a: *p* < 0.05 compared with the normal group. b: *p* < 0.05 compared with the ischemia group.

To investigate the regulatory effect of Drp1 on ER‐Mito contact and its subsequent impact on mitochondrial fission and function in vivo, we generated Drp1 knockout mice (Drp1+/‐) (Figure S4A–E, Supporting Information). TEM observations revealed that Drp1 knockout under normal conditions did not significantly affect ER‐Mito contact (*p* > 0.05), which could be attributed to the lower frequency of ER‐Mito contact in normal conditions (**Figure** **4**A; Figure S4F, Supporting Information). However, after ischemic injury, knockout of Drp1 significantly reduced the formation of ER‐Mito contacts and decreased the number of fragmented mitochondria in the superior mesenteric artery (SMA) tissue (*p* < 0.05; Figure 4B). Additionally, we examined the effects of Drp1 knockout on ER‐Mito contact formation in other vital organs, namely the heart, liver, and kidneys; TEM images indicated that Drp1 knockout also reduced ER‐Mito contact formation and mitochondrial fragmentation caused by excessive fission after ischemia (*p* < 0.05; Figure 4C; Figure S4F, Supporting Information). Furthermore, Drp1 knockout significantly improved mitochondrial function after ischemia (*p* < 0.05; Figure 4D). These findings confirm that Drp1‐initiated ER‐Mito contact is a prerequisite for excessive mitochondrial fission and dysfunction in vivo.

To investigate the specific mechanism by which Drp1 initiates ER‐Mito contact formation, we conducted sequencing of Drp1‐knockout mice (Drp1 KO, Drp1+/‐) and wild‐type mice (WT, Drp1+/+) (data update: GSE124096). The expression of the cytoskeleton regulatory protein Shrm4 was significantly reduced after Drp1 knockout (*p* < 0.05; Figure 4E). Shrm4 reportedly interacts directly with F‐actin and promotes F‐actin binding,^[^ ^15^ ^]^ which may contribute to the actin‐mediated traction anchoring process between the ER and mitochondria (Figure 4F). Western blotting results showed that Shrm4 expression was significantly increased after ischemic injury (*p* < 0.05), and was significantly reduced after Drp1 knockout (*p* < 0.05; Figure 4G). Furthermore, we found that the binding ability between Drp1 and Shrm4 was significantly enhanced at ischemia 0.5 h (*p* < 0.05; Figure 4H), consistent with the time point at which Drp1‐initiated ER‐Mito contact formation occurs. The structural analysis also identified potential binding sites between Drp1 and Shrm4 (The interaction free energy between Drp1 and Shrm4 is −18.9 kcal mol^−1^), supporting the notion that activated Drp1 can form a complex with Shrm4 (Figure 4I). These findings suggest that the formation of ER‐Mito contacts initiated by Drp1 may be related to the promotion of F‐actin binding and the enhancement of traction anchoring between the ER and mitochondria through via recruitment of Shrm4.

### 2.3 Drp1‐Mediated Shrm4 Recruitment to Induce Actin Bundling for ER‐Mito Contact Formation

**Figure 5:**
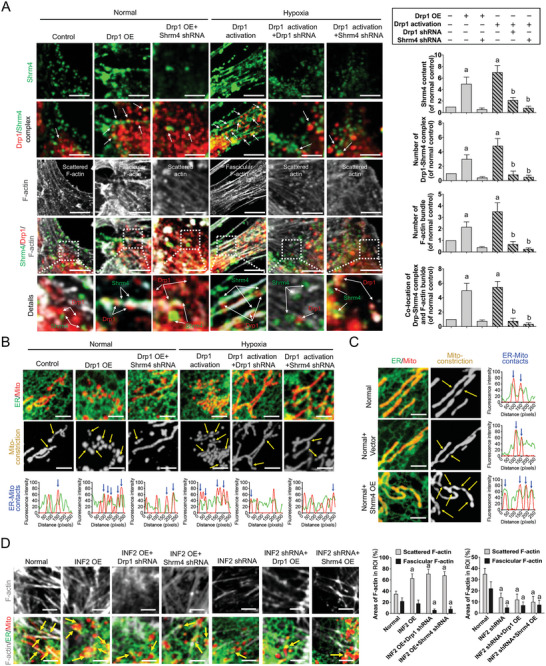
Influence of Drp1, Shrm4, and INF2 intervention on F‐actin bundling and ER‐Mito contact formation in VSMCs after hypoxia. A) Shrm4 expression, Drp1‐Shrm4 interaction, and F‐actin bundling in hypoxia‐induced VSMCs after altering Drp1 or Shrm4 expression. B) Representative confocal images of ER‐Mito contacts in hypoxia‐induced VSMCs after altering Drp1 or Shrm4 expression (bar, 5 µm). Yellow arrows: mitochondrial constriction sites. The co‐location of ER and Mitochondria (black arrows) are calculated by Image J. C) Representative confocal images of ER‐Mito contacts in normal VSMCs after Shrm4 overexpression (OE) (bar, 5 µm). Yellow arrows refer to mitochondrial constriction sites. The co‐locations of ER and Mitochondria (black arrows) are calculated by Image J. D) Representative confocal images of ER‐Mito contacts and F‐actin bundling in VSMCs after intervening Drp1, Shrm4, and INF2 (bar, 5 µm). Area percent of scattered F‐actin and fascicular F‐actin are calculated by Image J. a: *p* < 0.05 compared with the normal group. b: *p* < 0.05 compared with the Drp1 activation group.

To investigate the specific mechanism by which Shrm4 participates in Drp1‐initiated ER‐Mito contact formation after ischemia and hypoxia, we observed ER‐Mito contact formation and actin bundling through Shrm4 deletion or overexpression at the cellular level. Confocal images showed that both Drp1 overexpression and hypoxia‐induced Drp1 activation increased the number of Drp1‐Shrm4 complexes (*p* < 0.05) and induced the transformation of scattered F‐actin into fascicular F‐actin (**Figure** **5**A). Disrupting the formation of the Drp1‐Shrm4 complex by Shrm4 or Drp1 deletion blocked the transition from scattered F‐actin to fascicular F‐actin (Figure 5A) and reduced the number of ER‐Mito contacts and mitochondrial constriction (Figure 5B; Figure S5A, Supporting Information). Shrm4 overexpression increased mitochondrial constriction caused by ER‐Mito contact (*p* < 0.05; Figure 5C; Figure S5B, Supporting Information). These results suggest that Drp1 forms a complex by recruiting Shrm4, promotes actin bundling, enhances the traction anchoring between the ER and mitochondria, and initiates ER‐Mito contact formation.

Previous studies have shown that INF2, located at the ER, also plays a role in ER‐Mito contact formation.^[^ ^16^, ^17^ ^]^ To explore whether INF2 is involved in the Drp1‐Shrm4 complex‐initiated ER‐Mito contact formation, we observed the number of ER‐Mito contacts and actin bundling after different interventions with INF2, Drp1, and Shrm4 at the cellular level. Confocal results showed that INF2 overexpression alone significantly increased the number of scattered F‐actin (*p* < 0.05), but did not significantly change the number of fascicular F‐actin and ER‐Mito contact (*p* > 0.05) (Figure 5D). However, INF2 overexpression, while disrupting the Drp1‐Shrm4 complex, resulted in a significant decrease in the number of fascicular F‐actin and ER‐Mito contact (*p* < 0.05) (Figure 5D). Interestingly, regardless of the intervention in the formation of the Drp1‐Shrm4 complex, INF2 deletion resulted in a significant decrease in the number of scattered F‐actin, fascicular F‐actin, and ER‐Mito contact (*p* < 0.05) (Figure 5D). These results suggest that the formation of ER‐Mito contact and mitochondrial fission only occur when Drp1 recruits Shrm4 to induce actin bundling and requires the structural basis of INF2 linking with scattered F‐actin on ER.

### 2.4 Improved Multi‐Organ Function and Prognosis by Reducing ER‐Mito Contact Formation After Ischemic Injury Through DRP1 Intervention

**Figure 6:**
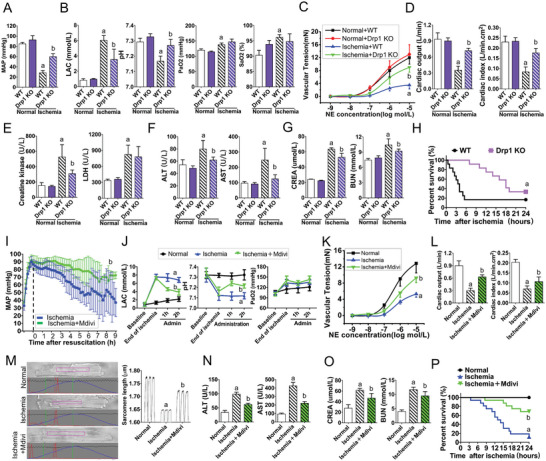
Intervention with Drp1 improves multi‐organ function and prognosis by reducing ER‐Mito contact formation after ischemic injury. A) Mean arterial pressure (MAP) of wild‐type and Drp1‐knockout mice in normal and ischemic conditions (*n* = 8 per group). B) Blood gas indexes, including LAC, pH, PaO_2_ and SaO_2_, of wild‐type and Drp1‐knockout mice in normal and ischemic conditions (*n* = 8 per group). C) Vascular reactivity of SMAs from wild‐type and Drp1‐knockout mice in normal and ischemic conditions (*n* = 8 per group). D) Cardiac functions (cardiac output and cardiac index) of wild‐type and Drp1‐knockout mice in normal and ischemic conditions (*n* = 8 per group). E) Creatine kinase and LDH values of wild‐type and Drp1‐knockout in normal and ischemic conditions (*n* = 8 per group). F) Liver functions (ALT and AST) of wild‐type and Drp1‐knockout mice in normal and ischemic conditions (*n* = 8 per group). G) Kidney functions (CREA and BUN) of wild‐type and Drp1‐knockout mice in normal and ischemic conditions (*n* = 8 per group). H) The 24‐h survival curves of wild‐type and Drp1‐knockout mice after ischemic injury (*n* = 16 per group). I) MAP monitoring of ischemic rats after treatment with Mdivi (*n* = 16 per group). J) Blood gas indexes including, LAC, pH, and PaO_2_, in ischemic rats after treatment with Mdivi (*n* = 8 per group). K) Statistical analysis of vascular reactivity in SMA tissues to NE stimulation in ischemic rats after treatment with Mdivi (*n* = 8 per group). L) Cardiac functions (cardiac output and cardiac index) in ischemic rats after treatment with Mdivi (*n* = 8 per group). M) Cellular contractility of acute isolated myocardial cells in ischemic rats after treatment with Mdivi. Sarcomere lengths calculated based on video (*n* = 5 per group). N) Liver function (ALT and AST) in ischemic rats after treatment with Mdivi (*n* = 8 per group). O) Kidney function (CREA and BUN) in ischemic rats after treatment with Mdivi (*n* = 8 per group). P) The 24‐h survival curves of ischemic rats after treatment with Mdivi (*n* = 16 per group). a: *p* < 0.05 compared with the normal or wild‐type group. b: *p* < 0.05 compared with the ischemic or ischemic wild‐type group.

To investigate the clinical relevance of Drp1‐mediated ER‐Mito contact formation, Drp1‐knockout mice and the Drp1 inhibitor, Mdivi, were employed to modulate the expression and activity of Drp1, respectively, and evaluate multi‐organ function and survival after ischemic injury. The findings demonstrated that Drp1‐knockout mice had favorable maintenance of mean arterial pressure (MAP) after ischemic injury (**Figure** **6**A) and that the levels of blood gas indicators, such as lactate and pH, were significantly improved compared to those in WT mice (*p* < 0.05; Figure 6B). Although there was no notable difference in the organ functions between Drp1‐knockout mice and WT mice under normal conditions (*p* > 0.05), Drp1 knockout mice exhibited better maintenance of organ function than WT mice after ischemic injury. This was primarily evidenced by the improvement of vasoconstriction tension recovery (*p* < 0.05; Figure 6C), enhanced cardiac output, cardiac index, other cardiac function indicators (*p* < 0.05; Figure 6D,E), and enhanced liver and kidney functions (*p* < 0.05; Figure 6F,G). Despite the 24‐h survival rate of Drp1‐knockout mice being only slightly higher than that of WT mice (33.3% versus 20%, *p* < 0.05), the overall survival time of Drp1‐knockout mice (17.50 ± 5.92 h) was significantly longer than that of wild mice (6.58 ± 8.27 h) (*p* < 0.05; Figure 6H). These findings indicate that modulating Drp1 expression can reduce ER‐Mito contact and protect multiple‐organ functions, ultimately improving the prognosis following ischemic injury.

Furthermore, we conducted experiments using Mdivi to inhibit Drp1 activity in rats with ischemic injury. Our results showed that blood pressure was maintained well (*p* < 0.05; Figure 6I), blood gas indicators improved (*p* < 0.05; Figure 6J), and vascular and cardiac function recovered to varying degrees (*p* < 0.05; Figure 6K,L). Additionally, myocardial cell contractility significantly increased (*p* < 0.05; Figure 6M; Movies [Link], [Link], [Link], Supporting Information), and liver and kidney function recovered (*p* < 0.05; Figure 6N,O). Moreover, the survival time and survival rate of rats with ischemic injury were significantly improved (*p* < 0.05; Figure 6P). These results provide further evidence that interventions targeting Drp1 can protect multiple‐organ function and improve prognosis after ischemic injury by reducing ER‐Mito contact.

## 3 Discussion

DRP1 is a crucial molecule that regulates mitochondrial fission. Previous studies have shown that ER winding to mitochondria occurs to form ER‐Mito contacts and mitochondrial constriction before Drp1‐mediated mitochondrial fission.^[^ ^18^, ^19^ ^]^ The question of whether Drp1 is involved in ER‐Mito contact formation and mitochondrial constriction has remained unanswered. Previous studies have shown that although 50% of total Drp1 is present in the mitochondria, only ≈14% of total DRP1 participates in mitochondrial fission, with 36% performing an unknown function in the outer membrane of mitochondria and the remaining 50% thought to have unknown cytoplasmic functions.^[^ ^20^ ^]^ Additionally, previous studies have reported that DRP1 has multiple modification sites leading to various types of modifications, such as phosphorylation, ubiquitination, and acetylation.^[^ ^21^, ^22^ ^]^ However, most of these modifications cannot be directly linked to their functional regulation. Meanwhile, in this study, we discovered that activated cytoplasmic Drp1 participates in ER‐Mito contact formation before translocating to mitochondria to mediate mitochondrial fission. Additionally, no previous studies have focused on the time course of Drp1 activation, ER‐Mito contact formation, mitochondrial fission, and mitochondrial function, which have traditionally been assumed to occur simultaneously. In our study, we utilized a systematic time course method to demonstrate that Drp1 modifications occur before mitochondrial fission, primarily through phosphorylation and S‐nitrosylation modifications of cytoplasmic Drp1, which establish a chemical foundation for Drp1‐initiated ER‐Mito contact formation. Importantly, Drp1 activation and ER‐Mito contact formation occurred before excessive mitochondrial fission and mitochondrial dysfunction during hypoxia‐induced mitochondrial quality imbalance.

ER‐Mito contact plays an important role in various aspects of mitochondrial quality control, including mitochondrial metabolism,^[^ ^23^ ^]^ calcium transfer,^[^ ^24^ ^]^ and mitochondrial dynamics,^[^ ^25^ ^]^ among others. Although the phenomenon of mitochondrial constriction in ER‐Mito contact was first described by Friedman JR et al.^[^ ^8^ ^]^ highlighting its role in mitochondrial fission, the question of whether this process is related to the function of activated Drp1 remained unclear. Nevertheless, Lewis SC et al.^[^ ^19^ ^]^ reported that ER‐Mito contact can contribute to the coupling of mtDNA replication with mitochondrial fission, regulating the biological function of mitochondria. This finding expanded the physiological role of ER‐Mito contact, suggesting that it can regulate mitochondrial physiological activities at multiple levels. Although several studies have investigated how ER‐Mito contact leads to mitochondrial fission,^[^ ^23^, ^26^ ^]^ the formation process and regulatory mechanism of ER‐Mito contact is unclear. Several linker proteins, including MFN2, PACS, and IP3R, have been identified in the ER‐Mito contact region that stabilize the relationship between the ER and mitochondria via their transmembrane domains and non‐covalent binding between proteins.^[^ ^27^, ^28^ ^]^ However, an in‐depth characterization of the physiological and pathological significance and mechanisms of these proteins under stress is lacking.^[^ ^29^ ^]^ Although traditionally thought to function only after ER‐Mito contact formation, Drp1 has primarily been evaluated in terms of how it translocates to mitochondria to mediate mitochondrial fission and fragmentation.^[^ ^30^, ^31^ ^]^ In this study, we discovered that Drp1 participates in the formation of ER‐Mito contacts. More specifically, the overexpression or activation of Drp1 activates Shrm4, resulting in the clustering of F‐actin into bundles and leading to ER‐Mito contact formation and mitochondrial constriction. Conversely, Drp1 knockout or inhibition decreases ER‐Mito contact formation and mitochondrial constriction. Hence, our results suggest that Drp1 is not only the main regulator of mitochondrial fission but also an important inducer of ER‐Mito contact formation and mitochondrial constriction.

The formation of ER‐Mito contact is closely related to INF2‐mediated actin polymerization.^[^ ^17^ ^]^ ER‐bound INF2 and the nearby polymerized F‐actin have unique functions. Korobova F et al. reported that INF2‐CAAX located on the ER can polymerize G‐actin in the ER‐Mito region, suggesting that this region of the polymerized actin plays an important role in the formation of mitochondrial constriction.^[^ ^32^ ^]^ Indeed, INF2‐CAAX clusters primarily at the ER‐Mito contact site and is regulated by numerous intracellular signals (such as Ca^2+^). Its main function involves polymerizing G‐actin to F‐actin, which is located in the gap formed between the mitochondria and ER. Under myosin pull, the lateral stress of F‐actin is applied to the mitochondria to cause mitochondrial constriction.^[^ ^9^, ^17^, ^33^ ^]^ However, the mechanism by which F‐actin promotes the wrapping of the ER around mitochondria to initiate ER‐Mito contact remained unclear. In this study, we found that Drp1 forms a complex with Shrm4. Overexpression or activation of Drp1 can activate Shrm4, which bundles F‐actin around mitochondria. The bundled F‐actin promotes ER migration and wrapping around mitochondria to form ER‐Mito contact sites and subsequent mitochondrial constriction. Meanwhile, disruption of the Drp1‐Shrm4 complex reduces the formation of F‐actin bundles and ER‐Mito contact. Moreover, the binding between INF2 and actin was sufficiently stable. Hence, given that the ER is reportedly more flexible than the mitochondria,^[^ ^34^ ^]^ we speculate that the structural stability formed by the Shrm4‐actin‐INF2 complex is sufficient to drive ER migration.

Shrm4 is a regulatory protein of the actin cytoskeleton that can promote the aggregation of F‐actin into actin bundles.^[^ ^35^ ^]^ Despite lacking an actin‐binding domain, as opposed to the Shrm2 and Shrm3 family members, which can bind actin directly, Shrm4 may regulate actin assembly via a synergistic effect with myosin.^[^ ^35^ ^]^ Moreover, certain myosins are closely associated with mitochondria, including myosin 19,^[^ ^35^ ^]^ whereas myosin II is present in the ER‐Mito contact site,^[^ ^35^ ^]^ and plays an important role in mitochondrial movement, constriction, and fission. Further investigation is needed to determine whether these myosins are involved in Shrm4‐mediated actin bundling and movement.

This study has certain limitations. First, we conducted time course observations and mechanistic exploration in VSMCs and vascular tissue, based on our previous studies,^[^ ^10^, ^12^, ^13^ ^]^ which revealed that VSMCs are relatively sensitive and representative of changes in mitochondrial quality after ischemia and hypoxia injury. We have also consistently observed ER‐Mito contact in other tissues, such as the heart, liver, and kidneys of Drp1‐knockout mice. Nevertheless, we plan to verify these conclusions in cells from other tissues in the future. Second, we only manipulated the expression of Shrm4 at the cellular level to observe the formation of ER‐Mito contacts. Although we initially intended to use Shrm4‐knockout mice for overall verification, embryonic death occurred, which may be related to the greater impact of regulating cytoskeleton layout on embryo development. In the future, we will further explore the construction of Shrm4 conditional knockout mice to facilitate more in‐depth investigations. Third, we solely confirmed the effect of the interaction between Drp1 and Shrm4 on the formation of ER‐Mito contacts without investigating the precise binding sites of Drp1 and Shrm4, or their interactions with actin, myosin, and INF2. Therefore, in our future research, we plan to explore these aspects further.

## 4 Conclusions

**Figure 7:**
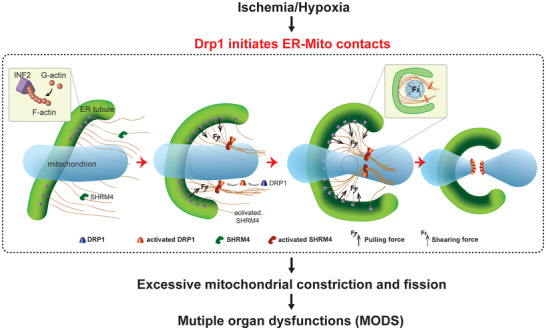
Schematic diagram showing activated Drp1 initiating ER‐Mito contacts via Shrm4‐mediated actin bundling after ischemia and hypoxia.

In this study, we investigated the temporal sequence of changes in mitochondrial quality, including Drp1 activation, ER‐Mito contact formation, mitochondrial fission, and mitochondrial function. Our findings suggest that cytoplasmic Drp1 participates in the formation of ER‐Mito contacts before translocating to the mitochondria to mediate mitochondrial fission. This process involves the recruitment of Shrm4 by activated Drp1 to induce actin bundling between the ER and mitochondria, promoting ER wrapping around mitochondria to form ER‐Mito contact. The process requires the structural basis of INF2 linking with scattered F‐actin on the ER (**Figure** **7**). Collectively, our findings broaden the understanding of the role of Drp1, identifying a potential target for regulating mitochondrial function and offering new insights for preventing and treating mitochondrial quality imbalances resulting from ischemia and hypoxia.

## 5 Experimental Section

### Animals

The Sprague‐Dawley rats used in the study were procured from the Research Institute of Surgery, Army Medical University (Chongqing, China, No. AMUWEC20171288), and all animal‐related procedures were approved by the Research Council and Animal Care and Use Committee of the same institute. The study adhered to the protocols outlined in the Guide for the Care and Use of Laboratory Animals (National Institutes of Health, Publication No. 85‐23, Revised 1996).

Drp1‐knockout C57BL/6J mice (Drp1 +/‐) were generated using the CRISPR/Cas9 technology (Shanghai Model Organisms Center, Inc. Shanghai, CHINA). Guide RNAs targeting intron 1 (5'–3': GCCATAAACCAGAGATGTCTGGG) and intron 2 (5'–3': TCGCTCATTTGGATTACACACGG) were designed, and exon 2 was removed based on the previous study.^[^ ^11^ ^]^ Founder lines of successful mutations in the Drp1 gene cluster were identified via PCR genotyping of tail DNA. The PCR products were further verified by DNA sequencing. The genotyping primers were as follows: Forward primer was 5'‐GAGTTGGTGAGGGAGGAGTGAGTA‐3', and the reverse primer was 5'‐CTGGGCATATAAGCAAGGTGA‐3'. The positive founder and WT male mice were bred to obtain F1 Drp1 heterozygous mice.

### Materials

Antibodies against Drp1 and β‐actin were purchased from Abcam (Cambridge, MA, USA), whereas those against Tubulin and COX IV were used as internal references for cytoplasmic and mitochondrial fractions, and were also purchased from Abcam. Pan‐antibodies for phosphorylation (Phospho‐) and S‐nitrosylation (SNO‐) modification, as well as antibodies for Shrm4, were purchased from Abclonal Technology (Wuhan, CHINA). ERTracker Green and MitoTracker Deep Red were purchased from Invitrogen (Carlsbad, CA, USA). Adenoviral vectors labeled with GFP, BFP, and RFP for Drp1 and Shrm4, as well as GFP‐tagged and BFP‐tagged Drp1 deletion (shDrp1, 1.5 ×10^7^ PFU), Drp1 overexpression (Drp1 OE, 3.0 ×10^7^ PFU), Shrm4 deletion (shShrm4, 1.5 ×10^7^ PFU), Shrm4 overexpression (Shrm4 OE, 3.0 ×10^7^ PFU), INF2 deletion (shINF2, 1.5 ×10^7^ PFU), and INF2 overexpression (INF2 OE, 3.0 ×10^7^ PFU) were generated by GeneChem Technology (Shanghai, CHINA). The ROS assay kit (DCFH‐DA), mitochondrial membrane potential assay kit with JC‐1, and ATP assay kit were purchased from Beyotime Biotechnology (Shanghai, CHINA). The Drp1 inhibitor, Mdivi, was purchased from BioTek (Winooski, VT, USA). Protein G agarose was purchased from Thermo Scientific (Waltham, MA, USA), and fetal bovine serum and penicillin/streptomycin were purchased from Invitrogen (Carlsbad, CA, USA). All other chemicals were purchased from Sigma unless otherwise specified.

### Model Preparation

To prepare the hypoxia model, VSMCs were incubated in a hypoxia compartment that was bubbled with hypoxic gas (95% N_2_/5% CO_2_) at 3 L min^−1^ for 15 min, followed by a 10‐min rest period. This procedure was repeated five times until the O_2_ concentration dropped below 0.2%. The cells were then cultured under hypoxic conditions for the specified duration of time (0.5 h, 1, 2, 3, or 4 h).^[^ ^10^ ^]^ To prepare the ischemia model, rats (220–240 g) and mice (18–25 g) were anesthetized with sodium pentobarbital (initial dosage of 30 mg kg^−1^). The rats and mice were placed on a warm plate to maintain their body temperature at 37 °C, and aseptic techniques were used for all surgical procedures. The right femoral arteries were catheterized with polyethylene catheters to withdraw 50% of total blood volume (≈7% of weight). Times were calculated after the model was established and maintained for various durations (0.5, 1, 2, 3, or 4 h).^[^ ^13^ ^]^ After ischemia, animals were euthanized with a lethal dose of sodium pentobarbital (100 mg kg^−1^, iv). A laparotomy was performed to obtain the SMA and digest it into VSMCs. Meanwhile, ischemia rats received a continuous infusion of Mdivi (1 mg kg^−1^), as previously reported.^[^ ^13^ ^]^

### Confocal Imaging and Video Observation

For confocal imaging, Leica STELLARIS (Leica Microsystems, Wetzlar, Germany) inverted confocal microscopes were used fitted with a 60 × 1.3 NA oil‐immersion objective for imaging at 37 °C inside incubators. The pinhole was adjusted to maintain a thickness of optical slices at 2 µm. Mitochondria in cells were stained with MitoTracker Deep Red (100 nm for 30 min, 37 °C), which was excited using a 633 nm laser, and emission was collected at 558–617 nm. ER in cells were labeled with ERTracker Green (500 nm for 30 min, 37 °C), which was excited using a 488 nm laser, and emission was collected at 501–563 nm. For confocal video observation, the acquisition mode was selected as “x‐y‐t” and time‐lapse 2D images were captured at a speed of 15 s per frame to observe mitochondrial dynamic changes, constriction, and fission.

### Transmission Electronic Microscopy Imaging

Fresh tissues were fixed with an arsenate buffer containing 2.5% glutaraldehyde for 24 h at 4 °C and pH 7.4. After three 10‐min washes with 0.13 M PBS, the tissues were post‐fixed with 1% OsO_4_ for 2 h at 25 °C and then dehydrated in a graded series of ethanol (65%, 70%, 75%, 80%, and 95%, each for 10 min). Next, the tissues were incubated with tert‐butoxide for 10 min and dried with CO_2_. They were then stained with uranyl acetate or lead citrate, coated with gold (Au) using an ion sputter coater, and viewed and imaged with TEM (H‐7500, Hitachi Company, Japan).^[^ ^3^ ^]^

### 3D Reconstruction with FIB‐SEM

Samples of SMAs were cut into small blocks of ≈1 mm and fixed in a solution containing 2.5% glutaraldehyde and 2% paraformaldehyde. The blocks were then immersed in 0.2 m PBS buffer (pH 7.4) at room temperature (≈25 °C) for 2 h. After washing, the samples were stained with reduced osmium tetroxide‐thiocarbohydrazide (TCH) osmium (ROTO) and infiltrated with Epon.^[^ ^36^ ^]^ Serial sections with a thickness of 8 nm were obtained using focused ion beam scanning electron microscopy (FIB‐SEM) (Version: Helios NanoLab G3UC, Thermo Fisher Scientific, Hillsboro, Oregon, USA). The software AMIRA 6.2.0 (Thermo Fisher Scientific, Hillsboro, Oregon, USA) was used to stack, align, and model FIB‐SEM tomograms of mitochondria and ER.

### Subcellular Fractionation

Isolated SMAs or VSMCs were collected in filter cartridges, and cytosolic fractions were isolated using a Minute Cytoplasmic Extraction Kit (Invent Biotechnologies, Inc. SC‐003).^[^ ^37^ ^]^ Mitochondrial fractions were isolated using a Minute Mitochondria Isolation Kit (Invent Biotechnologies, Inc. MP‐007).^[^ ^38^ ^]^ The fractionated proteins were then used for immunoblotting analyses with the indicated antibodies.

### Vascular Tension Detection

The vasoconstriction response to norepinephrine (NE) was evaluated using an isolated organ perfusion system (Scientific Instruments, Barcelona, Spain). The procedure involved cutting each SMA into 1–2 mm rings, which were then suspended between a force transducer and immersed in an isolated organ chamber (Scientific Instruments, Barcelona, Spain) containing 5 mL Krebs–Henseleit solution (K–H solution). After a 5‐min equilibration period, the contractile response was determined by the cumulative administration of NE. The tension of the artery rings was recorded via a force transducer (AD Instruments)^[^ ^13^ ^]^ using a Power Lab System.

### Immunoprecipitation (IP) and Western Blotting

Protein G IP Kit (Roche, Switzerland) was used for IP according to the manufacturer's instructions. For western blotting, cell pellets were lysed with RIPA buffer (Beyotime Institute of Biotechnology, China) containing complete Protease Inhibitors (Roche, Switzerland) and PhosSTOP Phosphastase Inhibitors (Roche, Switzerland), electrophoresed, then blotted onto PVDF membranes. The membranes were incubated with the indicated primary antibodies, followed by incubation with HRP‐conjugated secondary antibodies (Jackson ImmunoResearch, UK). The protein concentration was calculated using the BCA Protein assay kit (Thermo Scientific Pierce, UK). Blotted proteins were visualized using an enhanced chemiluminescence detection kit (Tiangen Biotech, China), and the intensity of the bands was analyzed using Quantity One V 4.62 Software (Bio‐Rad, Life Science, California, USA).

### Statistics Analysis

The results were presented as means and standard deviations. For experiments with more than two groups, one‐way analysis of variance was performed, followed by Tukey's post hoc analysis. Kaplan–Meier survival analysis was conducted using SPSS 17.0 (SPSS Inc., Chicago, IL, USA). A significance level of *p* < 0.05 was used to indicate statistical significance.

## Conflict of Interest

The authors declare no conflict of interest.

## Author Contributions

D.C.Y. performed most of the experiments in this study and wrote the manuscript. D.C.Y., L.T., and L.L.M. designed the whole project. L.R.X. and K.L. assisted in performing time course recording. Z.Z.S and H.D.Y. assisted in animal experiments. Z.D.Y. and X.X.M. assisted in cellular experiments. X.X.M. was in charge of instrument operation. D.C.Y., L.T., and H.H were responsible for manuscript editing and revision, and they provided scientific research funding support. All authors have read and approved the final manuscript.

## Supporting information

Supporting Information

Supplemental Movie 1

Supplemental Movie 2

Supplemental Movie 3

Supplemental Movie 4

Supplemental Movie 5

Supplemental Movie 6

Supplemental Movie 7

## Data Availability

The data that support the findings of this study are available on request from the corresponding author. The data are not publicly available due to privacy or ethical restrictions.

## References

[1] D. C. Chan , Annu. Rev. Pathol. 2020, 15, 235. 31585519 10.1146/annurev-pathmechdis-012419-032711

[2] F. Kraus , K. Roy , T. J. Pucadyil , M. T. Ryan , Nature 2021, 590, 57. 33536648 10.1038/s41586-021-03214-x

[3] X. Zeng , Y.‐D. Zhang , R.‐Y. Ma , Y.‐J. Chen , X.‐M. Xiang , D.‐Y. Hou , X.‐H. Li , H. Huang , T. Li , C.‐Y. Duan , Mil. Med. Res. 2022, 9, 25. 35624495 10.1186/s40779-022-00383-2 PMC9137164

[4] B. Haileselassie , R. Mukherjee , A. U. Joshi , B. A. Napier , L. M. Massis , N. P. Ostberg , B. B. Queliconi , D. Monack , D. Bernstein , D. Mochly‐Rosen , J. Mol. Cell. Cardiol. 2019, 130, 160. 30981733 10.1016/j.yjmcc.2019.04.006 PMC6948926

[5] C. Duan , R. Ma , X. Zeng , B. Chen , D. Hou , R. Liu , X. Li , L. Liu , T. Li , H. Huang , Front. Immunol. 2022, 13, 946731. 35844544 10.3389/fimmu.2022.946731 PMC9283956

[6] A. M. Bertholet , T. Delerue , A. M. Millet , M. F. Moulis , C. David , M. Daloyau , L. Arnauné‐Pelloquin , N. Davezac , V. Mils , M. C. Miquel , M. Rojo , P. Belenguer , Neurobiol. Dis. 2016, 90, 3. 26494254 10.1016/j.nbd.2015.10.011

[7] J.‐P. Leduc‐Gaudet , S. N. A. Hussain , E. Barreiro , G. Gouspillou , Int. J. Mol. Sci. 2021, 22, 8179. 34360946 10.3390/ijms22158179 PMC8348122

[8] J. R. Friedman , L. L. Lackner , M. West , J. R. Dibenedetto , J. Nunnari , G. K. Voeltz , Science 2011, 334, 358. 21885730 10.1126/science.1207385 PMC3366560

[9] F. Korobova , V. Ramabhadran , H. N. Higgs , Science 2013, 339, 464. 23349293 10.1126/science.1228360 PMC3843506

[10] C. Duan , L. Kuang , C. Hong , X. Xiang , J. Liu , Q. Li , X. Peng , Y. Zhou , H. Wang , L. Liu , T. Li , Cell Death Dis. 2021, 12, 1050. 34741026 10.1038/s41419-021-04343-x PMC8571301

[11] C. Duan , L. Kuang , X. Xiang , J. Zhang , Y. Zhu , Y. Wu , Q. Yan , L. Liu , T. Li , Aging 2020, 12, 1397. 31954373 10.18632/aging.102690 PMC7053642

[12] C. Duan , L. Kuang , X. Xiang , J. Zhang , Y. Zhu , Y. Wu , Q. Yan , L. Liu , T. Li , Cell Death Dis. 2020, 11, 251. 32312970 10.1038/s41419-020-2461-9 PMC7170874

[13] C. Duan , L. Wang , J. Zhang , X. Xiang , Y. Wu , Z. Zhang , Q. Li , K. Tian , M. Xue , L. Liu , T. Li , Redox Biol. 2020, 37, 101706. 32911435 10.1016/j.redox.2020.101706 PMC7490562

[14] J. Steffen , C. M. Koehler , J. Cell Biol. 2018, 217, 15. 29259094 10.1083/jcb.201711075 PMC5748997

[15] M. Yoder , J. D. Hildebrand , Cell Motil. 2007, 64, 49. 10.1002/cm.20167 17009331

[16] M. A , T. S. Fung , L. M. Francomacaro , T. Huynh , T. Kotila , Z. Svindrych , H. N. Higgs , Proc. Natl. Acad. Sci. USA 2020, 117, 439. 31871199 10.1073/pnas.1914072117 PMC6955303

[17] R. Chakrabarti , W.‐K. Ji , R. V. Stan , J. De Juan Sanz , T. A. Ryan , H. N. Higgs , J. Cell Biol. 2018, 217, 251. 29142021 10.1083/jcb.201709111 PMC5748994

[18] K. B. Ackema , C. Prescianotto‐Baschong , J. Hench , S. C. Wang , Z. H. Chia , H. Mergentaler , F. Bard , S. Frank , A. Spang , PLoS One 2016, 11, 0154280. 10.1371/journal.pone.0154280 PMC4839682 27101143

[19] S. C. Lewis , L. F. Uchiyama , J. Nunnari , Science 2016, 353, aaf5549. 27418514 10.1126/science.aaf5549 PMC5554545

[20] B. M. Michalska , K. Kwapiszewska , J. Szczepanowska , T. Kalwarczyk , P. Patalas‐Krawczyk , K. Szczepanski , R. Holyst , J. Duszynski , J. Szymanski , Sci. Rep. 2018, 8, 8122. 29802333 10.1038/s41598-018-26578-z PMC5970238

[21] L. Tilokani , S. Nagashima , V. Paupe , J. Prudent , Essays Biochem 2018, 62, 341. 30030364 10.1042/EBC20170104 PMC6056715

[22] S. M. Adaniya , J. O‐Uchi , M. W. Cypress , Y. Kusakari , B. S. Jhun , Am. J. Physiol. Cell Physiol. 2019, 316, C583. 30758993 10.1152/ajpcell.00523.2018 PMC6580160

[23] G. Csordás , D. Weaver , G. Hajnóczky , Trends Cell Biol. 2018, 28, 523. 29588129 10.1016/j.tcb.2018.02.009 PMC6005738

[24] T. K. Acharya , A. Kumar , S. Kumar , C. Goswami , Life Sci. 2022, 310, 121112. 36283455 10.1016/j.lfs.2022.121112

[25] A. Markovinovic , J. Greig , S. M. Martín‐Guerrero , S. Salam , S. Paillusson , J. Cell Sci. 2022, 135, jcs248534. 35129196 10.1242/jcs.248534

[26] H. Mao , W. Chen , L. Chen , L. Li , Biochem. Pharmacol. 2022, 199, 115011. 35314166 10.1016/j.bcp.2022.115011

[27] B. Gottschalk , Z. Koshenov , O. A. Bachkoenig , R. Rost , R. Malli , W. F. Graier , Front. Cell Dev. Biol. 2022, 10, 918691. 36158213 10.3389/fcell.2022.918691 PMC9493370

[28] M. I. Hernández‐Alvarez , D. Sebastián , S. Vives , S. Ivanova , P. Bartoccioni , P. Kakimoto , N. Plana , S. R. Veiga , V. Hernández , N. Vasconcelos , G. Peddinti , A. Adrover , M. Jové , R. Pamplona , I. Gordaliza‐Alaguero , E. Calvo , N. Cabré , R. Castro , A. Kuzmanic , M. Boutant , D. Sala , T. Hyotylainen , M. Oresic , J. Fort , E. Errasti‐Murugarren , C. M. P. Rodrígues , M. Orozco , J. Joven , C. Cantó , M. Palacin , et al., Cell 2019, 177, 881. 31051106 10.1016/j.cell.2019.04.010

[29] R. Puri , X.‐T. Cheng , M.‐Y. Lin , N. Huang , Z.‐H. Sheng , Nat. Commun. 2019, 10, 3645. 31409786 10.1038/s41467-019-11636-5 PMC6692330

[30] J.‐Y. Jin , X.‐X. Wei , X.‐L. Zhi , X.‐H. Wang , D. Meng , Acta Pharmacol. Sin. 2021, 42, 655. 32913266 10.1038/s41401-020-00518-y PMC8115655

[31] Y. Wang , M. Lu , L. Xiong , J. Fan , Y. Zhou , H. Li , X. Peng , Z. Zhong , Y. Wang , F. Huang , W. Chen , X. Yu , H. Mao , Cell Death Dis. 2020, 11, 29. 31949126 10.1038/s41419-019-2218-5 PMC6965618

[32] V. Ramabhadran , F. Korobova , G. J. Rahme , H. N. Higgs , Mol. Biol. Cell 2011, 22, 4822. 21998196 10.1091/mbc.E11-05-0457 PMC3237625

[33] F. Korobova , T. J. Gauvin , H. N. Higgs , Curr. Biol. 2014, 24, 409. 24485837 10.1016/j.cub.2013.12.032 PMC3958938

[34] P. Georgiades , V. J. Allan , G. D. Wright , P. G. Woodman , P. Udommai , M. A. Chung , T. A. Waigh , Sci. Rep. 2017, 7, 16474. 29184084 10.1038/s41598-017-16570-4 PMC5705721

[35] O. A. Quintero , M. M. Divito , R. C. Adikes , M. B. Kortan , L. B. Case , A. J. Lier , N. S. Panaretos , S. Q. Slater , M. Rengarajan , M. Feliu , R. E. Cheney , Curr. Biol. 2009, 19, 2008. 19932026 10.1016/j.cub.2009.10.026 PMC2805763

[36] J. C. Tapia , N. Kasthuri , K. J. Hayworth , R. Schalek , J. W. Lichtman , S. J. Smith , J. Buchanan , Nat. Protoc. 2012, 7, 193. 22240582 10.1038/nprot.2011.439 PMC3701260

[37] H. Hu , W. Zhu , J. Qin , M. Chen , L. Gong , L. Li , X. Liu , Y. Tao , H. Yin , H. Zhou , L. Zhou , D. Ye , Q. Ye , D. Gao , Hepatology 2017, 65, 515. 27774669 10.1002/hep.28887

[38] G. Gao , Z. Wang , L. Lu , C. Duan , X. Wang , H. Yang , Biomed. Pharmacother. 2017, 96, 1380. 29169728 10.1016/j.biopha.2017.11.057

