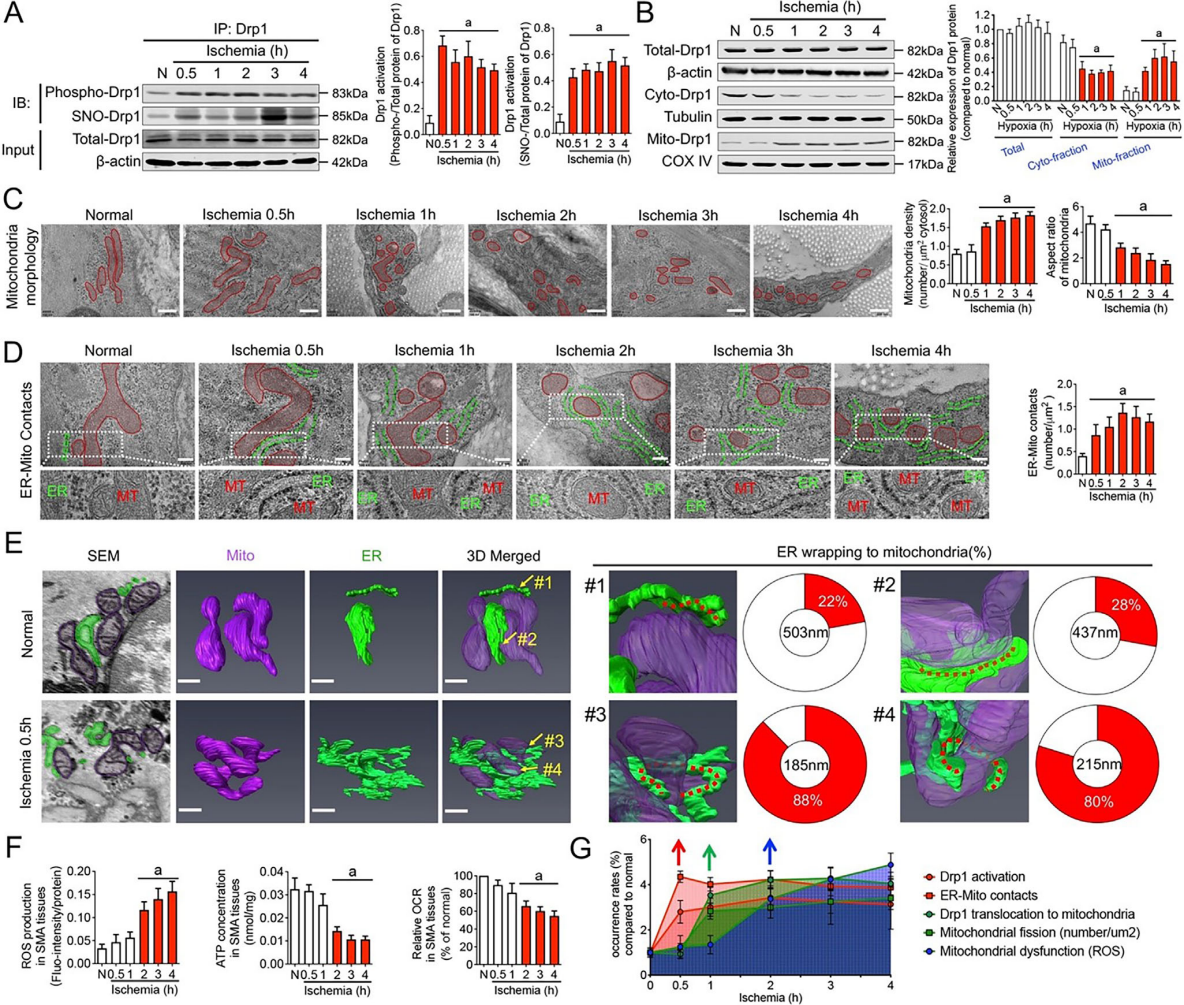# Correction to “Activated Drp1 Initiates the Formation of Endoplasmic Reticulum‐Mitochondrial Contacts via Shrm4‐Mediated Actin Bundling”

**DOI:** 10.1002/advs.202504531

**Published:** 2025-04-26

Duan C, Liu R, Kuang L, et al. Activated Drp1 Initiates the Formation of Endoplasmic Reticulum‐Mitochondrial Contacts via Shrm4‐Mediated Actin Bundling. *Adv Sci (Weinh)*. 2023;10(36):e2304885.

1. In Figure 5A, the magnification scale of the confocal images for the Hypoxia + Drp1 activation + Shrm4 shRNA group was inconsistent with the other groups. To ensure accurate data presentation, we have adjusted the scale accordingly. The revised Figure 5 is provided below.
2. In Figure 2B, the β‐actin loading control band in the Western blot (WB) was incorrectly used. We have replaced it with the correct band. Since the expression of target proteins was normalized using total protein quantification prior to analysis, this correction does not affect the scientific conclusions of the study. The revised Figure 2 is provided below.

We apologize for this error.

The revised Figure 5:


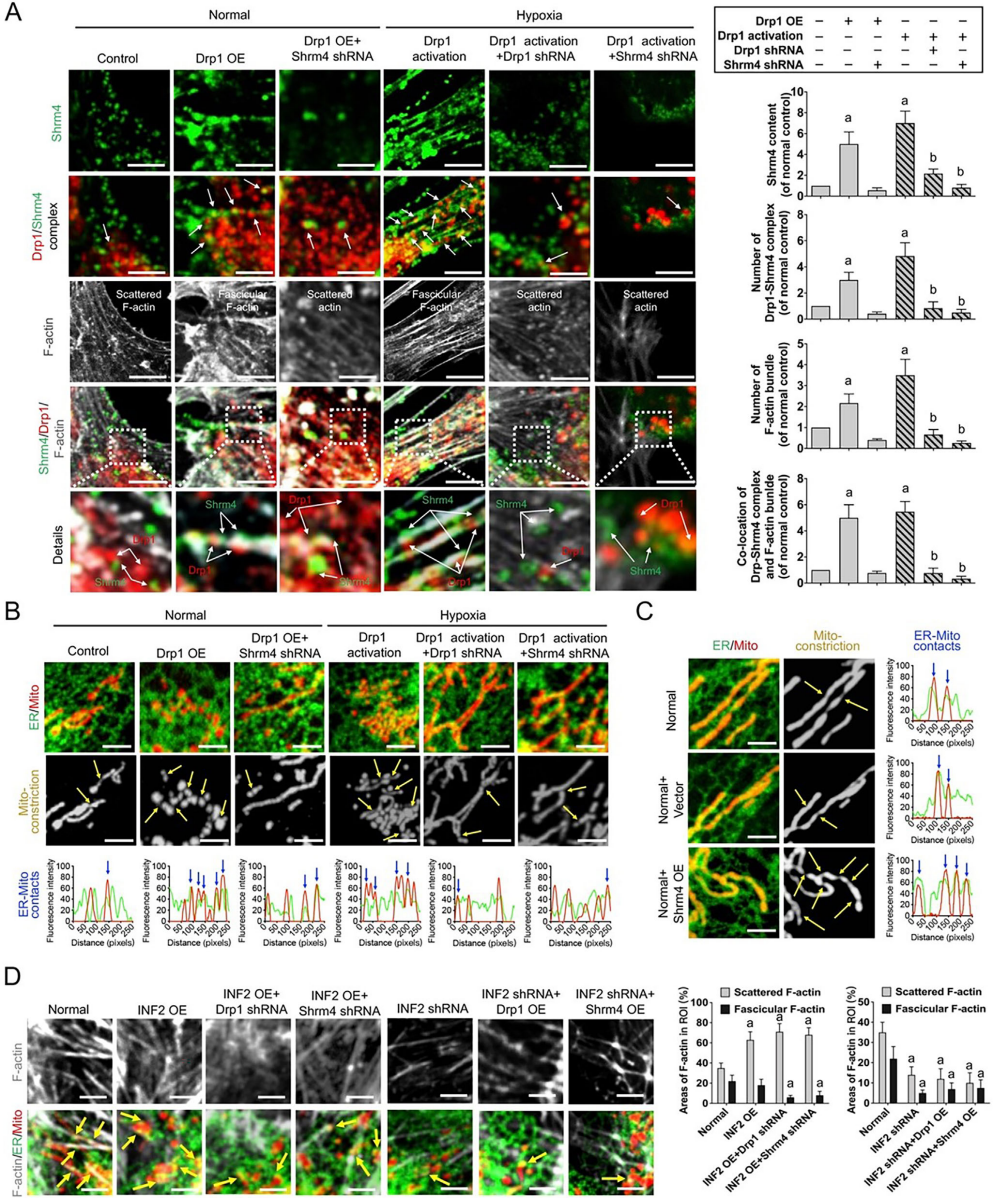


The revised Figure 2: