# Supplementary material for: Activated Drp1 Initiates the Formation of Endoplasmic Reticulum‐Mitochondrial Contacts via Shrm4‐Mediated Actin Bundling
Source: Adv Sci (Weinh). 2023 Nov 1;10(36):2304885. doi: 10.1002/advs.202304885 (PMC10754141; doi:10.1002/advs.202304885)
Supplement: Supplementary file 1 — Supporting Information [file ADVS-10-2304885-s008.pdf]

ADVANCED SCIENCE
Open Access

Supporting Information

for *Adv. Sci.*, DOI 10.1002/advs.202304885

Activated Drp1 Initiates the Formation of Endoplasmic Reticulum-Mitochondrial Contacts via Shrm4-Mediated Actin Bundling

Chenyang Duan, Ruixue Liu, Lei Kuang, Zisen Zhang, Dongyao Hou, Danyang Zheng, Xinming Xiang, He Huang, Liangming Liu* and Tao Li**

Supporting Information

Activated Drp1 Initiates the Formation of Endoplasmic Reticulum-Mitochondrial Contacts via Shrm4-Mediated Actin Bundling

Chenyang Duan, Ruixue Liu, Lei Kuang, Zisen Zhang, Dongyao Hou, Danyang Zheng, Xinming Xiang, He Huang, Liangming Liu*, and Tao Li**

Figures

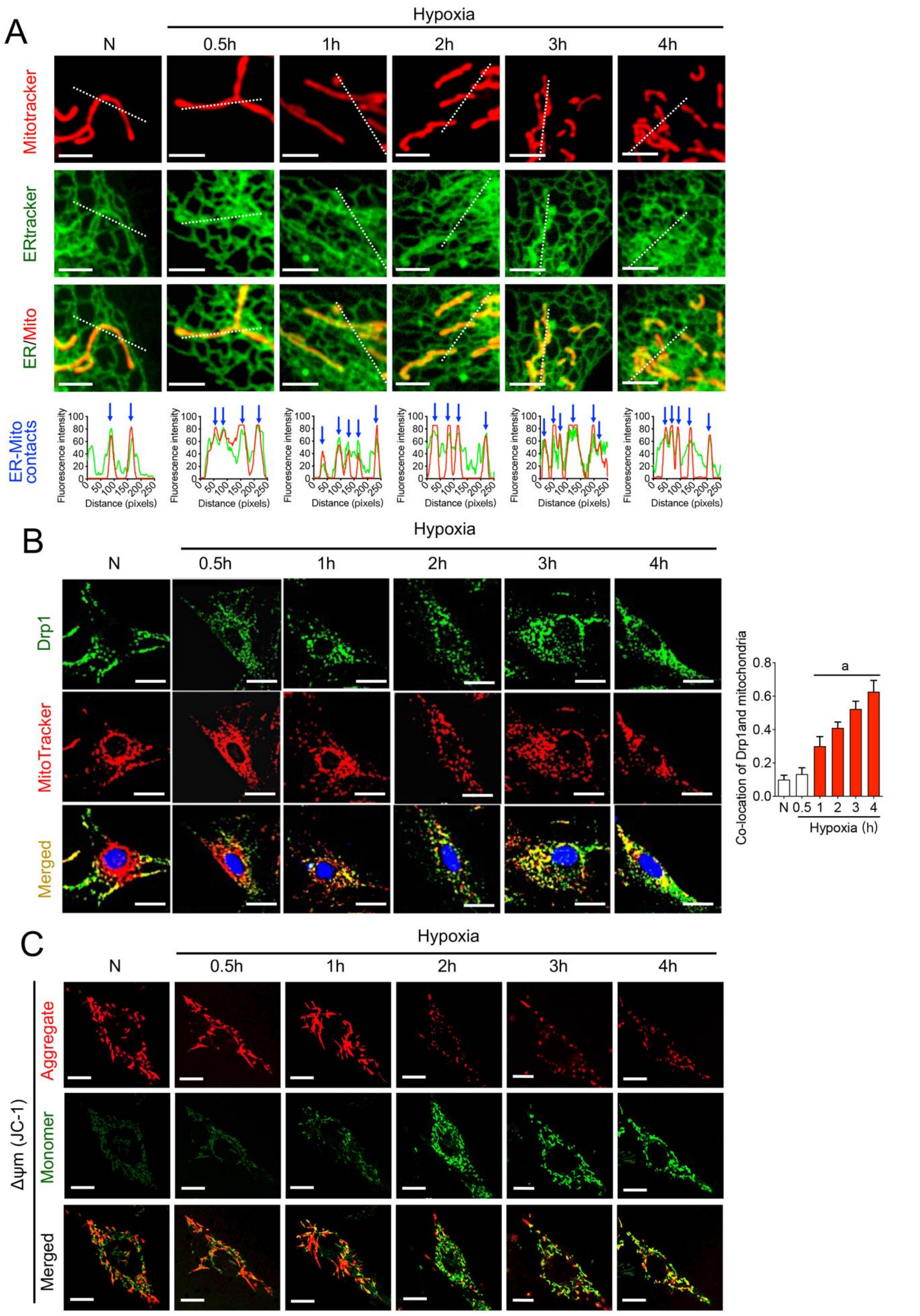

Figure S1. Time course observation of dynamin-related protein 1 (Drp1) translocation to mitochondria, and mitochondrial membrane potential in vascular smooth muscle cells

(VSMCs) with the extension of hypoxia (A) ER-Mito contact labeled with ERtracker (green) and Mitotracker (red) in VSMCs with the extension of hypoxia. White hatched lines: measurement path of Image J. Blue arrows: ER-Mito contacts along the measurement path. (B) Confocal images showing the co-location of Drp1 and mitochondria in VSMCs with the extension of hypoxia (bar, 25 μm). (C) Confocal images showing mitochondrial membrane potential labeled by JC-1 monomer (green fluorescent probe) and JC-1 aggregate (red fluorescent probe) in VSMCs with the extension of hypoxia (bar, 25 μm). a: $p < 0.05$ compared with the normal group.

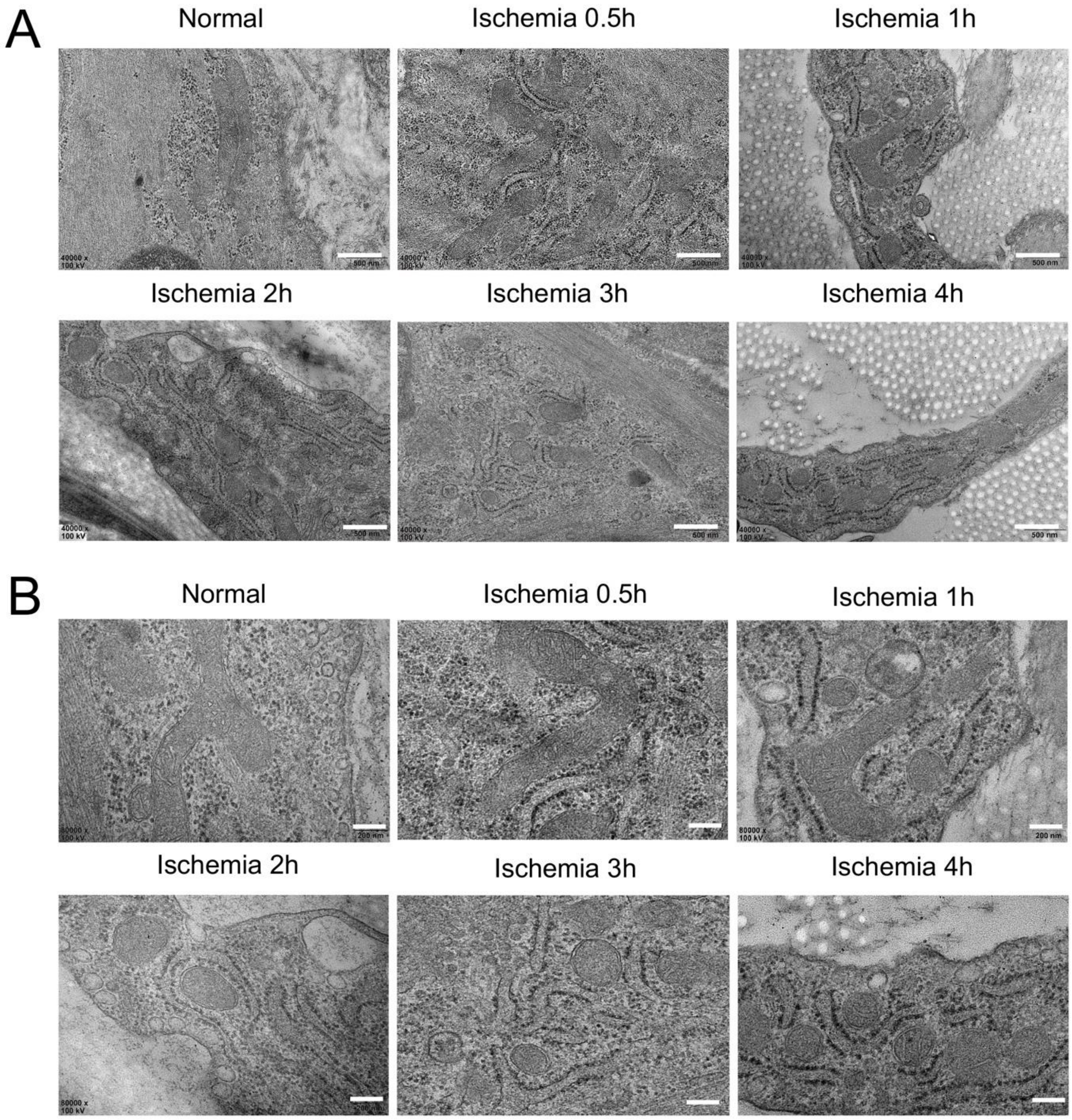

Figure S2. Original transmission electron microscopy (TEM) images of mitochondrial morphology and ER-Mito contacts in VSMCs with the extension of ischemia. (A) Unprocessed TEM images in Figure 2C of mitochondrial morphology in VSMCs with the extension of ischemia (bar, 500 nm). (B) Unprocessed TEM images in Figure 2D of ER-Mito contacts in VSMCs with the extension of ischemia (bar, 200 nm).

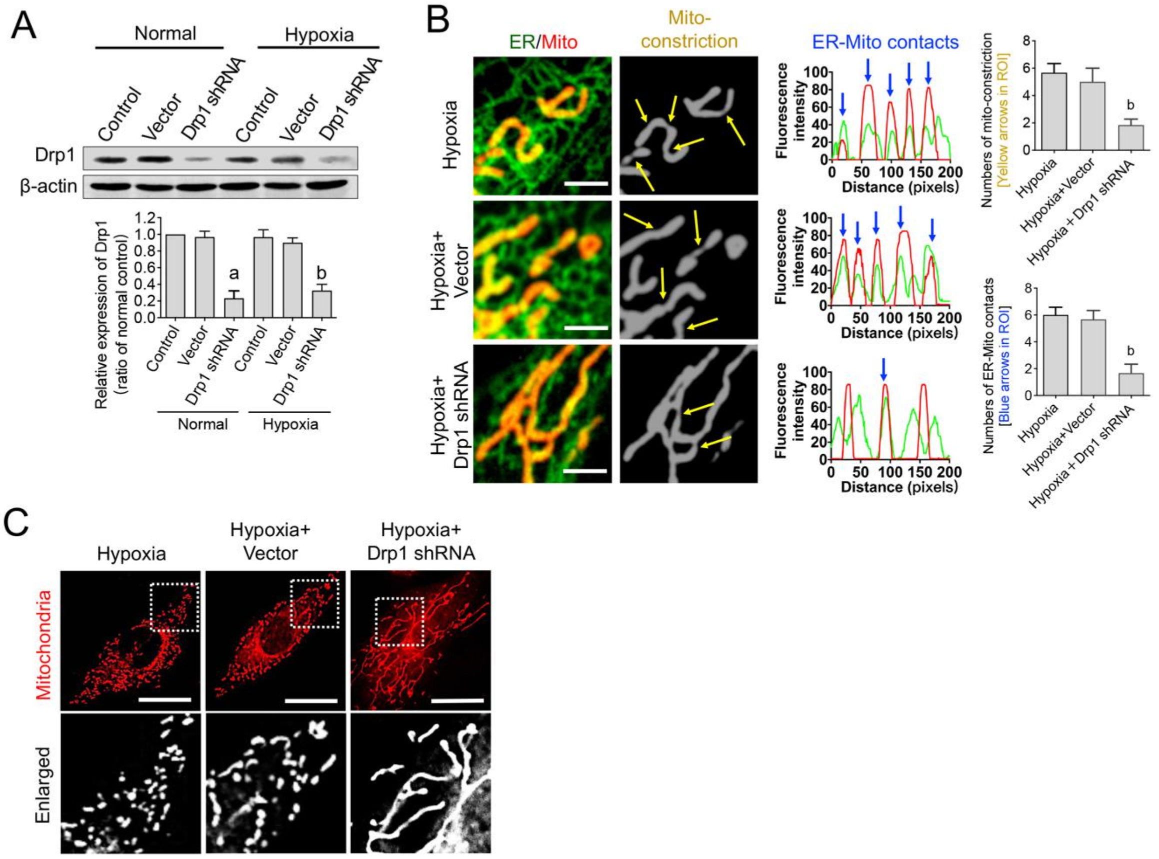

Figure S3. Influence of Drp1 deletion on ER-Mito contact and mitochondrial morphology in VSMCs after hypoxia (A) Western blotting results showing the effect of Drp1 deletion (Drp1 shRNA) on Drp1 protein expression in VSMCs in normal and hypoxia conditions (n = 3/group). (B) Representative confocal images of ER-Mito contacts labeled with ERtracker and Mitotracker in hypoxia-induced VSMCs after Drp1 deletion (bar, 5 μm). Yellow arrows: mitochondrial constriction sites. Blue arrows: ER-Mito contacts. Number of mitochondrial constriction and ER-Mito contacts in the region of interest were calculated by Image J (n = 5/group). (C) Representative confocal images of mitochondrial morphology in VSMCs. Confocal images recording mitochondrial morphology in hypoxia-induced VSMCs after Drp1 deletion (bar, 25 μm). a: $p < 0.05$ compared with the normal group. b: $p < 0.05$ compared with the hypoxia group.

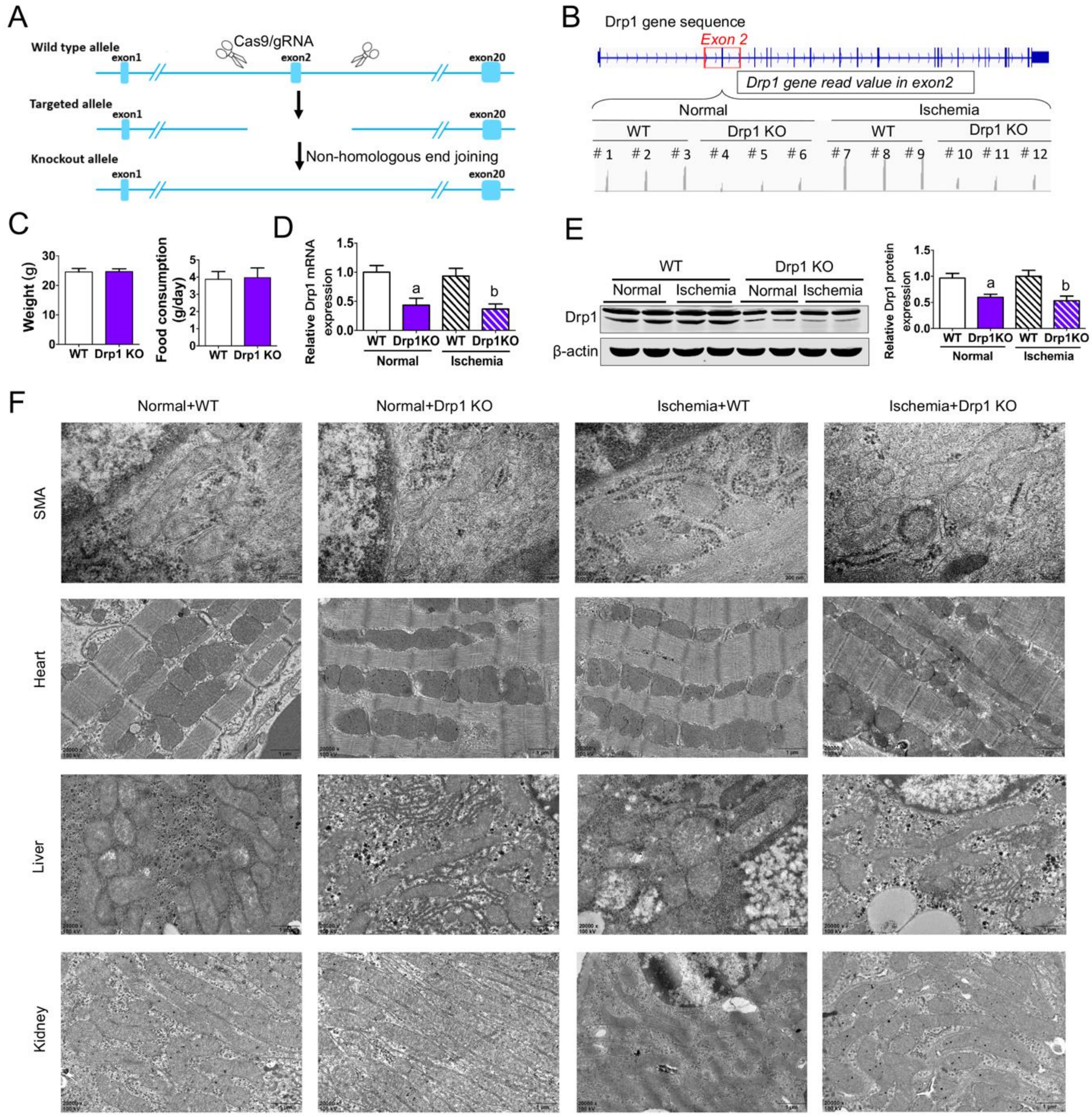

Figure S4. Verification of Drp1 effect in knockout mice and the original TEM images of ER-Mito contacts in various tissues from Drp1-knockout mice (A) Knockout strategy for DRP1-knockout mice (*Drp1*+/-). (B) Sequencing results showing reduced exon 2 read value in DRP1-knockout mice. (C) Weight and food consumption of wild-type and DRP1-knockout mice in normal conditions. (D) DRP1 mRNA expression in superior mesenteric arteries (SMAs) from wild-type and DRP1-knockout mice in normal and ischemic conditions determined via qRT-PCR. (E) DRP1 protein expression in SMAs from wild-type and DRP1-knockout mice in normal and ischemic conditions determined by Western blotting. (F) Unprocessed TEM images

in Figure 4 of ER-Mito contacts in SMA, heart, liver, and kidneys from wild-type and Drp1-knockout mice in normal and ischemic conditions. a: $p < 0.05$ compared with the normal group. b: $p < 0.05$ compared with the ischemia group.

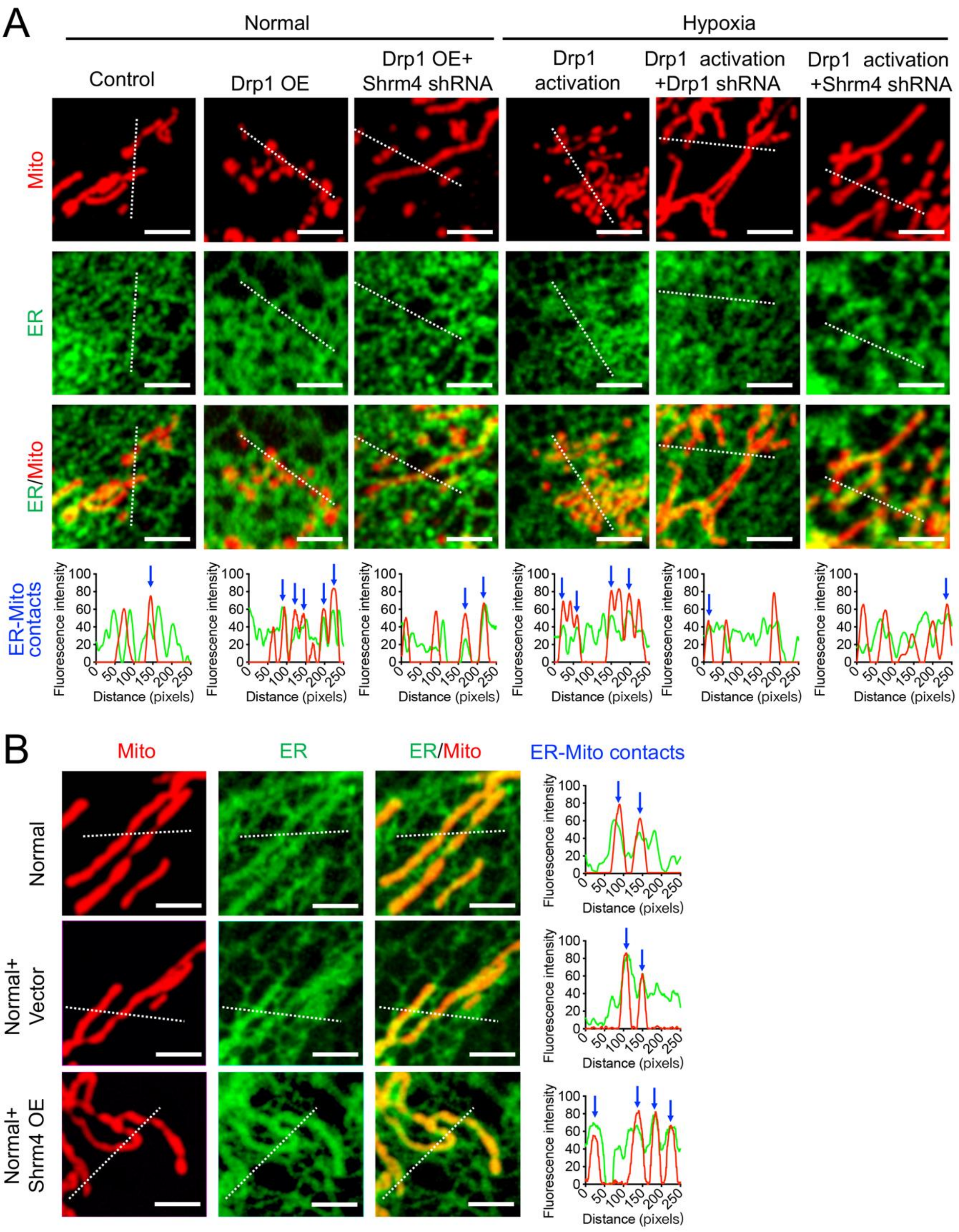

Figure S5. ER-Mito contacts in hypoxia-induced VSMCs after altering Drp1 or Shrm4 expression

(A) confocal images of ER-Mito contacts labeled with ERtracker (green) and Mitotracker (red) in hypoxia-induced VSMCs after altering Drp1 or Shrm4 expression (bar, 5 μm). White hatched

lines: measurement path of Image J. Blue arrows: ER-Mito contacts along the measurement path. (B) Confocal images of ER-Mito contacts labeled with ERtracker (green) and Mitotracker (red) in normal VSMCs after Shrm4 overexpression (OE) (bar, 5 μm). White hatched lines: measurement path of Image J. Blue arrows: ER-Mito contacts along the measurement path.

Expanded View Movies

Movie 1. Confocal video observation of mitochondrial fission under normal condition at the cellular level

Movie 2. Confocal video observation of excessive mitochondrial fission at hypoxia 1 h at the cellular level

Movie 3. Three-dimensional reconstruction of ER-Mito contact under normal condition at the animal level

Movie 4. Three-dimensional reconstruction of ER-Mito contact after ischemia 1 h at the animal level

Movie 5. Contractility recording of acute isolated myocardial cells from a normal rat

Movie 6. Contractility recording of acute isolated myocardial cells from an ischemic rat

Movie 7. Contractility recording of acute isolated myocardial cells from an ischemic rat after treatment with Mdivi
